# Activation of Cofilin Increases Intestinal Permeability *via* Depolymerization of F-Actin During Hypoxia *in vitro*

**DOI:** 10.3389/fphys.2019.01455

**Published:** 2019-12-03

**Authors:** Huapei Song, Jian Zhang, Wen He, Pei Wang, Fengjun Wang

**Affiliations:** Department of Burns, State Key Laboratory of Trauma, Burns, and Combined Injury, Institute of Burn Research, Southwest Hospital, Army Medical University, Chongqing, China

**Keywords:** hypoxia, intestinal epithelial cells, permeability, cofilin, actin, tight junction

## Abstract

Mechanical barriers play a key role in maintaining the normal function of the intestinal mucosa. The barrier function of intestinal epithelial cells is significantly damaged after severe hypoxia. However, the molecular mechanisms underlying this hypoxia-induced damage are still not completely clear. Through the establishment of an *in vitro* cultured intestinal epithelial cell monolayer model (Caco-2), we treated cells with hypoxia or drugs [jasplakinolide or latrunculin A (LatA)] to detect changes in the transepithelial electrical resistance (TER), the expression of the cellular tight junction (TJ) proteins zonula occludens-1 (ZO-1) and occludin, the distribution of F-actin, the ratio of F-actin/G-actin content, and the expression of the cofilin protein. The results showed that hypoxia and drug treatment could both induce a significant reduction in the TER of the intestinal epithelial cell monolayer and a significant reduction in the expression of the ZO-1 and occludin protein. Hypoxia and LatA could cause a significant reduction in the ratio of F-actin/G-actin content, whereas jasplakinolide caused a significant increase in the ratio of F-actin/G-actin content. After hypoxia, cofilin phosphorylation was decreased. We concluded that the barrier function of the intestinal epithelial cell monolayer was significantly damaged after severe burn injury. The molecular mechanism might be that hypoxia-induced F-actin depolymerization and an imbalance between F-actin and G-actin through cofilin activation resulted in reduced expression and a change in the distribution of cellular TJ proteins.

## Introduction

The intestine has always been considered the main organ impacted by surgical stress, such as trauma from severe burns. Damage to the barrier function of the intestine is an important factor in pathophysiological disorders of the body after severe burn injury (Osuka et al., 2017; He et al., 2019). Mechanical, chemical, immunological, and biological barriers together constitute the normal intestinal mucosal barriers, of which the mechanical barrier is the most important. The key to the function of the mechanical barrier is the function of tight junction (TJ) barriers. TJs mainly include occludin, zonula occludens family members, junctional adhesion molecules, and claudin family members. TJs are regulated by many factors, including Ca^2+^-E-cadherin and Rho-GTPase (Blikslager et al., 2007; Rübsam et al., 2017). Cell retraction is an important link between barrier function damage and increased permeability in intestinal epithelial cells. It is both regulated by myosin and influenced by actin. F-actin in the cytoskeleton and myosin forms an actin-myosin ring to participate in cell retraction. Under normal conditions, F-actin and G-actin maintain a dynamic balance. However, when this balance is broken, the barrier function is damaged (Turner, 2009). It has been shown that severe burn injury causes TJ damage in intestinal epithelial cells and reduces intestinal barrier functions. Changes in F-actin in the intestinal epithelial cytoskeleton, as well as intracellular TJ proteins, may be involved in the occurrence of intestinal epithelial barrier function damage (Chen et al., 2012). However, the specific molecular mechanisms are still not clear.

Cofilin and actin depolymerizing factor (ADF) belong to the actin-binding protein family (Maciver and Hussey, 2002). Their basic functions are in the depolymerization of F-actin and the inhibition of G-actin polymerization, which play critical roles in remodeling cytoskeletal microfilaments and retraction regulation (Pfaendtner et al., 2010; Wang et al., 2010). It has been shown that the cofilin upstream protein Slingshot (SSH) could cause the dephosphorylation of phosphorylated Ser3 on cofilin to induce cofilin activation (Terry et al., 2010; Wang et al., 2015). However, whether changes in the cofilin activity participate in intestinal barrier function damage after hypoxia is currently not clear.

## Materials and Methods

### Materials

The Caco-2 cell line was purchased from ATCC. Dulbecco’s modified Eagle medium (DMEM), nonessential amino acid (NEAA), and fetal bovine serum (FBS) were purchased from GIBCO (Thermo Fisher Scientific, MA, United States), and cell culture incubator and multiplate reader were purchased from Thermo Fisher (Thermo Fisher Scientific, Waltham, MA, United States). Trypsin was purchased from BBI Life Sciences Corporation (Shanghai, China). Twenty-four-well plate Transwell chambers (8 mm per well) were purchased from Corning (Corning, NY, United States). The Alexa Fluor 488-deoxyribonuclease and Alexa Fluor 594-phalloidin were purchased from Invitrogen (Thermo Fisher Scientific, Waltham, MA, United States). TCS SP5 laser confocal microscope was purchased from Leica (Germany). Anti-β-actin, anti-cofilin, anti-phosphorylated cofilin, anti-ZO-1, anti-occludin, and FITC-dextran fluorescence probe were purchased from Sigma-Aldrich (St. Louis, MO, United States). Polyvinylidene fluoride (PVDF) membrane and the Millicell ERS voltohmmeter were purchased from Millipore (EMD Millipore, Billerica, MA, United States). Jasplakinolide and latrunculin A (LatA) were purchased from Calbiochem (United States). UD-201 tissue and cell ultrasonicator were purchased from Tomy (Japan). RC DC protein assay kit, protein assay kit, protein electrophoresis reagents, electrophoresis cell, electroporation devices, and ChemiDoc XRS gel imaging system were purchased from Bio-Rad Laboratories (Hercules, CA, United States).

### Cell Culture

The Caco-2 cells were cultured in DMEM (pH 7.4), which contains 100 U/ml penicillin, 100 mg/ml streptomycin, 10% FBS, 2 mmol/L glutamine, and 1 mmol/L NEAA at 37°C in an incubator containing 5% CO_2_ and saturated humidity. The medium was replaced every other day until 80% confluence, followed by partial digestion with Ca^2+^-free and Mg^2+^-free Hank’s balanced saline solution containing 0.25% trypsin and 0.53 mmol/L EDTA. The cells were subcultured in a ratio of 1:3.

The cells were inoculated into 96-well plates and onto the polycarbonate membrane cell culture chambers of 24-well Transwell plates at a density of 5 × 10^4^ cell/cm^2^, onto coverslips in 24-well plates at a density of 2.5 × 10^5^ cell/cm^2^, and into six-well plates at a density of 5 × 10^5^ cell/cm^2^. The conditions of cell growth in six-well plates, on coverslips in 24-well plates, and in 96-well plates were observed every day under an inverted microscope.

### Cell Treatment

#### Hypoxia Treatment

Caco-2 cells were seeded overnight in plates, followed by treatment with hypoxia. They were randomly divided into six groups using a random number table. Hypoxia was induced according to previously published methods (Wang et al., 2013). Cells were treated with hypoxia for 0 (no treatment), 1, 2, 6, 12, and 24 h. The hypoxia conditions were 1% O_2_, 5% CO_2_, and a 94% N_2_ mixed gas environment.

#### Drug Treatment

Caco-2 cells were seeded overnight in plates, followed by treatment with drugs. They were randomly divided into six groups using a random number table. Jasplakinolide was prepared at a final concentration of 0.1 μmol/L and was added to the cells for treatments for 0 (no treatment), 0.25, 0.5, 1, 2, 6, and 24 h. Using the same method, Lat A was prepared at a final concentration of 0.2 μmol/L and was added to the cells for treatment for 0 (immediately), 0.25, 0.5, 1, 2, 6, and 24 h.

### Transepithelial Electrical Resistance Assay

The Caco-2 cells were seeded in 24-well Transwell plates to grow as monolayers. After hypoxia and drug treatments, the Millicell ERS voltohmmeter (Millipore, United States) was used to measure the TER. To clarify the difference of TER between treatment groups and nontreatment group (0 h) more clearly, the normalization method was used to express TER ratio of treatment group to nontreatment group (0 h).

### Caco-2 Cell Permeability Assay

FITC-dextran fluorescence probe tracing was used to detect the permeability of the Caco-2 cell monolayers pretreated with drugs for 24 h. The specific manipulation was as follows: the culture medium in the Transwell was discarded, and the upper and lower chambers of the Transwell were washed using D-Hank’s solution. Next, 500 μl of D-Hank’s solution was added into the lower chamber, and 100 μl of the FITC-dextran solution was added into the upper chamber. Cells were routinely cultured for 2 h in an incubator. The fluorescence intensity of the solution in the lower chamber was detected using a multifunctional plate reader with excitation and emission wavelengths of 490 and 520 nm, respectively. The normalization method was used to express the fluorescence intensity ratio of treatment group to nontreatment group (N).

### Indirect Immunofluorescence Assay

Caco-2 cells were collected at different time points after hypoxia treatment. The culture medium was discarded, and PBS was added into the 24-well Transwell and washed for 5 min. The PBS was then discarded, and an appropriate amount of 4% paraformaldehyde was added for fixation at the room temperature for 30 min. The polycarbonate membrane of the Transwell was washed with PBS for 5 min and rinsed in PBS containing 50 mmol/L NH4Cl at room temperature for 15 min. The membrane was then washed three times for 5 min with PBS. Next, 2.5% BSA (prepared in PBS) was added for blocking at room temperature for 30 min. The PBS was discarded, and the primary anti-ZO-1 antibody at a dilution of 1:200 or the primary anti-occludin antibody at a dilution of 1:100 was added in 50 μl/well and incubated overnight in a 4°C refrigerator. The membrane was then washed again with PBS (three times, 5 min). DAPI stain at a dilution of 1:2,000 mixed with a Texas red-labeled secondary antibody at a dilution of 1:50 or an Alexa Fluor 488-labeled secondary antibody at a dilution of 1:100 was added in 50 μl/well and incubated at room temperature for 1 h in the dark. The membrane was then washed with PBS (three times, 5 min). The polycarbonate Transwell membrane was cut along the edge using a razor blade, rinsed in ddH_2_O for 10 s, and placed onto a slide. Next, 2.5 μl of Slowfade solution was dropped onto the slide and covered with a coverslip. The slide was observed under a TCS SP5 laser scanning confocal microscope and photographed.

### Actin Dynamics Assay

Caco-2 cells inoculated onto 96-well plates were collected at different time points after hypoxia. The culture medium in the original wells was discarded, and the Caco-2 cells in the 96-well plates were washed with PBS (three times, 5 min). The PBS was discarded, and an appropriate amount of 4% paraformaldehyde was added for fixation at the room temperature for 15 min. The cells were then washed with PBS (three times, 5 min). Next, 0.1% Triton X-100 was added, and the cells were incubated for 15 min at room temperature, after which 25 g/L BSA was added for blocking, and the cells were incubated at room temperature for 30 min. The solution was discarded, and the cells were washed with PBS (three times, 5 min). Alexa Fluor 594-phalloidin (final concentration of 0.165 mol/L) and Alexa Fluor 488-DNase I (final concentration of 0.3 mol/L) were added in 50 μl/well and incubated for 15 min to perform specific fluorescence labeling of F-actin and G-actin, respectively. Cells were washed with PBS again (three times, 5 min). The PBS was discarded, and the red fluorescence intensity (reflecting the relative F-actin levels) and green fluorescence intensity (reflecting the relative G-actin levels) were detected with excitation and emission wavelengths of 578 and 496 nm, and 600 and 519 nm, respectively. The ratio between the relative levels of F-actin and G-actin was the ratio between the intensity of red and green fluorescence.

### Fluorescence Probe Detection

Caco-2 cells in plates were collected at different time points after hypoxia treatment. The culture medium in the original wells was discarded, and the Caco-2 cells on coverslips were washed with PBS for 5 min. The PBS was discarded, and the Caco-2 cells were fixed in 4% paraformaldehyde at the room temperature for 15 min. The Caco-2 cells on coverslips were washed with PBS (three times, 5 min), and 0.1% Triton X-100 was added, followed by incubation at room temperature for 15 min. Next, 25 g/L BSA was added for blocking, and the cells were incubated at room temperature for 30 min. The BSA was discarded, and the coverslips were washed with PBS (three times, 5 min). Alexa Fluor 594-phalloidin (final concentration of 0.11 mol/L) was added in 50 μl/well and incubated at room temperature for 15 min. The coverslips were then washed with PBS (three times, 5 min). An appropriate amount of anti-fluorescence fade reagent was dropped onto the coverslips, and they were placed onto slides with the Caco-2 cells facing downward. Slides were observed under a laser scanning confocal microscope.

### Western Blot

These experiments were performed according to previously published methods using β-actin as an internal control (Cao et al., 2013; Song et al., 2018). Briefly, Caco-2 cells were inoculated into six-well culture plates. When the cells became confluent, they were treated with hypoxia or drugs and then cultured for an additional 24 h. The culture medium was discarded, and the cells were washed with cold PBS once. Then, cell lysis buffer was added, and the cells were collected and disrupted using an ultrasonic homogenizer. The cells were centrifuged at 4°C and 12,000 rpm for 10 min, and the supernatant was collected and boiled in water for 5 min. An equal amount of total proteins was subject to SDS-PAGE electrophoresis (Bio-Rad, USA) and transferred onto a PVDF membrane. The PVDF membrane was blocked in 5% skim milk for 1 h and incubated with corresponding primary antibodies (ZO-1, occludin, cofilin, p-cofilin, and the internal control β-actin) at 4°C overnight. After washing four times with TBST for 15 min, the membrane was incubated with corresponding secondary antibodies at room temperature for 1 h. The membrane was washed again four times with TBS, the chemiluminescent solution was added, and the chemiluminescent signals were collected using the ChemiDoc XRS system. The results were analyzed using Quantity One software.

### Statistical Analysis

SPSS17.0 (SPSS Inc., Chicago, IL, United States) and Excel 2013 (Microsoft, Redmond, WA, United States) software were used for statistical analysis. Data are expressed as the mean ± SD. The comparison of mean values between two samples was performed using the *t* test. The comparisons among groups were performed using the one-way analysis of variance (ANOVA). *p* < 0.05 indicated that the difference was significant and had statistical significance.

## Results

### Hypoxia Decreased the Transepithelial Electrical Resistance of Caco-2 Cells

**Figure 1:**
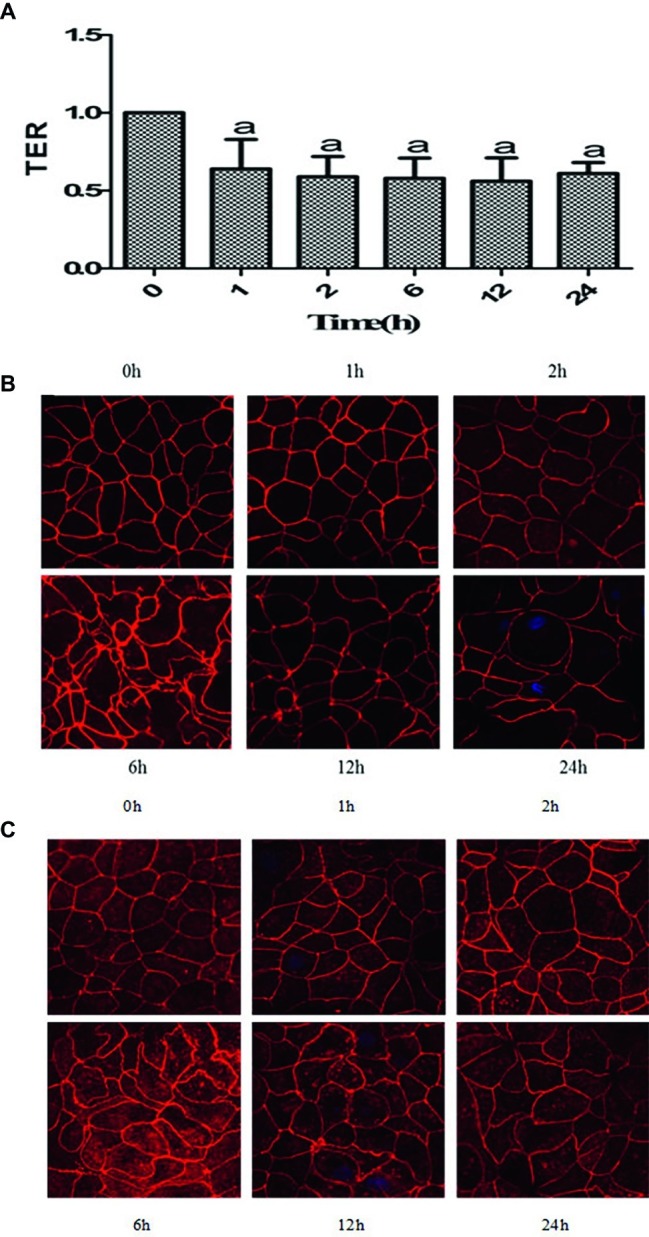
Hypoxia induced disruption of barrier function in Caco-2 cells. **(A)** Caco-2 cells were treated with hypoxia for 0, 1, 2, 6, 12, and 24 h, respectively. Transepithelial electrical resistance (TER) assay showed that the TER ratio of the intestinal epithelial cell monolayer decreased significantly after hypoxia, ^a^*p* < 0.05 compared with the control (0 h group). **(B,C)** Caco-2 cells were treated with hypoxia for 0, 1, 2, 6, 12, and 24 h, respectively. The proteins were labeled with Texas red. With increased exposure to hypoxia, zonula occludens (ZO)-1 **(B)** and occludin **(C)** both had irregular distributions and underwent changes, such as folds, serrations, and breaks. Scale bar = 10 μm. Data are representative of five similar experiments.

It is known that TER is a marker of epithelial barrier function. As shown in Figure 1A, compared to that at 0 h (before hypoxia), the TER ratio of the Caco-2 cell monolayer decreased significantly at different time points after hypoxia. Thus, it is indicated that the barrier function of Caco-2 cell monolayers was significantly disrupted after hypoxia.

### Hypoxia Disrupted Distributions of Zonula Occludens-1 and Occludin of Caco-2 Cells

Altered tight junction structure often contributes to the impaired epithelial barrier function. We further explored the effect of hypoxia on the distribution of the TJ proteins ZO-1 and occludin using immunofluorescence combined with confocal microscopy. The results showed that before hypoxia (0 h), the Caco-2 cells were densely arranged, with ZO-1 (Figure 1B) and occludin (Figure 1C) proteins exhibiting a smooth and continuous linear distribution along the cell membrane. With increased exposure to hypoxia over time, the cell morphology became very irregular with the cell bodies exhibiting obvious retraction and even showed gaps between cells. It is demonstrated that ZO-1 and occludin both had irregular distributions and underwent changes, such as folds, serrations, and breaks.

### Hypoxia Induced Disorganization of F-Actin Structure

**Figure 2:**
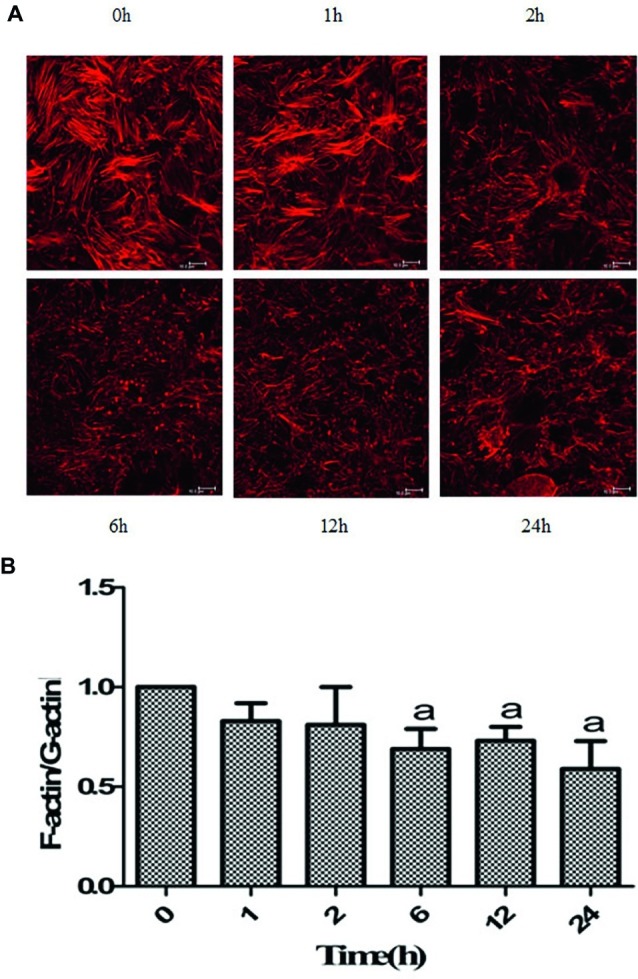
Hypoxia induced the disruption of actin networks. **(A)** Caco-2 cells were treated with hypoxia for 0, 1, 2, 6, 12, and 24 h, respectively. The F-actin filaments were labeled with Alexa Fluor 594-phalloidin. Fluorescence probe detection showed that the direction of the fibers in the cells became disordered, and the density of the intracellular F-actin fibers decreased after hypoxia. After 24 h of hypoxia, the F-actin filaments were obviously disrupted. Scale bar = 10 μm. **(B)** Caco-2 cells were treated with hypoxia for 0, 1, 2, 6, 12, and 24 h, respectively. The ratio between the relative levels of F-actin and G-actin at 0 h was set to 1. The F-/G-actin ratio was significantly decreased at 6, 12, and 24 h of hypoxia. ^a^*p* < 0.05 compared with 0 h group. Data are representative of five similar experiments.

The regulation of actin networks is critical to numerous physical cellular processes, including cell contraction, adhesion, migration, and division. Based on the abovementioned results, we then focused on F-actin in hypoxia-treated Caco-2 cells (Freischmidt et al., 2015). As shown in Figure 2A, the F-actin protein was very abundant in the cytoplasm of Caco-2 cells before hypoxia (0 h), forming a bundled shape. In addition, the fibers in the area of the cell nucleus were denser than the fibers at the cell edge. After hypoxia, the direction of the fibers in the cells became disordered, and the density of the intracellular F-actin fibers significantly decreased. Especially, treated with hypoxia for 24 h, the direction of the fibers was totally disordered, with no intact F-actin structure.

### Hypoxia Disrupted Actin Dynamics

It has been recognized that cell shape and mechanical force generation, to a large degree, are regulated by the dynamic mechanical behaviors of a diverse assortment of actin networks and bundles (Stricker et al., 2010; Salter et al., 2016). The ratios were reduced to different degrees in Caco-2 cells after hypoxia exposure, depending on the time points. The ratios at 6, 12, and 24 h of hypoxia were significantly lower than that before hypoxia (Figure 2B). Therefore, it is indicated that actin dynamics was disrupted after hypoxia stimulation.

### Jasplakinolide or Latrunculin A Decreased Transepithelial Electrical Resistance of Caco-2 Cells

**Figure 3:**
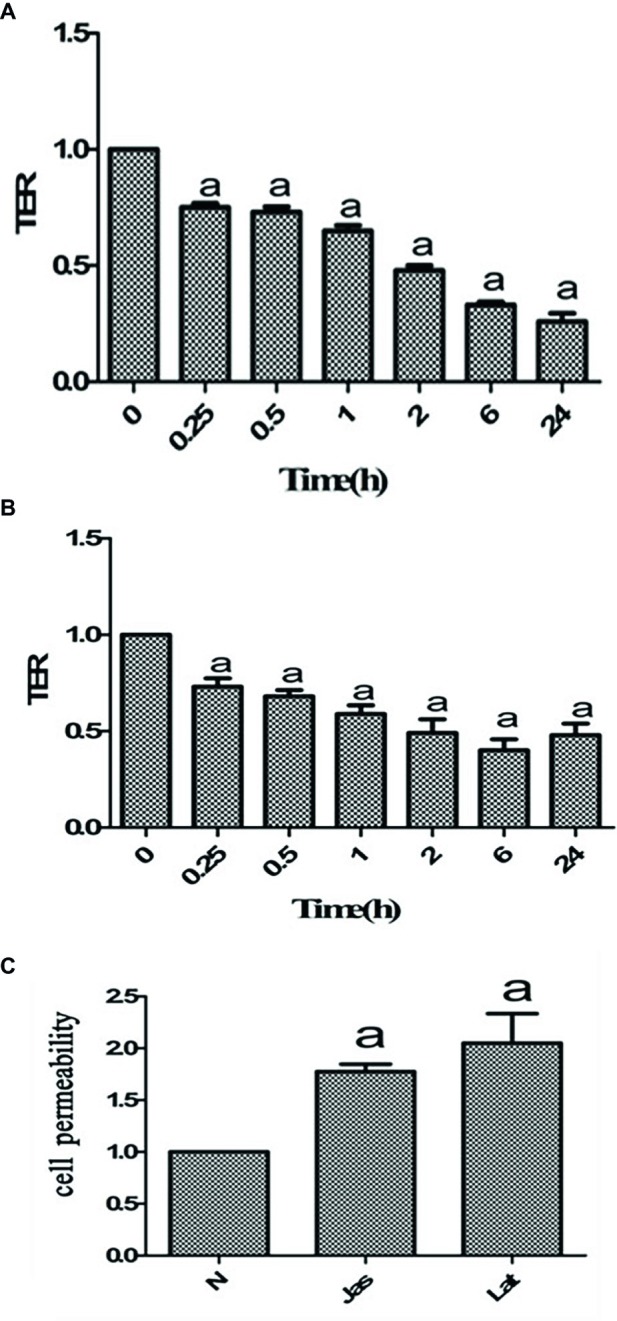
Pharmacological intervention with jasplakinolide or latrunculin A (LatA) disrupted the barrier function of Caco-2 cells. **(A,B)** Caco-2 cells were pretreated with jasplakinolide **(A)** or LatA **(B)** for 0, 0.25, 0.5, 1, 2, 6, and 24 h, respectively. The bar graphs showed a decrease in transepithelial electrical resistance (TER) ratio in Caco-2 cells, with significant reductions at different time points compared to that at 0 h. ^a^*p* < 0.05 compared with 0 h group. **(C)** Caco-2 cells were pretreated with jasplakinolide or LatA for 24 h. The FITC-dextran fluorescence assay showed the permeability of the Caco-2 cells significantly increased after pharmacological intervention. ^a^Compared with control group, *p* < 0.05. Data are representative of five similar experiments.

It is well known that jasplakinolide and LatA can both cause a deregulation of the F-actin/G-actin balance (Oliveira et al., 1996; Holzinger and Blaas, 2016; Pospich et al., 2017). After Caco-2 cell monolayers were pretreated with jasplakinolide or LatA, there was a gradual decreasing trend in the TER, with significant reductions at different time points compared to that at 0 h. These results indicated that both jasplakinolide (Figure 3A) and LatA (Figure 3B) treatment induced damage to the barrier function of Caco-2 cells.

### Jasplakinolide or Latrunculin A Increased Caco-2 Cell Permeability

Many studies have shown that cell permeability is associated with TER. We further investigated the effect of jasplakinolide or LatA on cell permeability. As shown in Figure 3C, compared to the control group, the FITC-dextran permeability of the Caco-2 cell monolayers was significantly increased after the addition of jasplakinolide or LatA. These results indicated that both jasplakinolide and LatA treatment induced significant increases in the permeability of Caco-2 cells.

### Jasplakinolide or Latrunculin A Decreased Zonula Occludens-1 and Occludin Level

**Figure 4:**
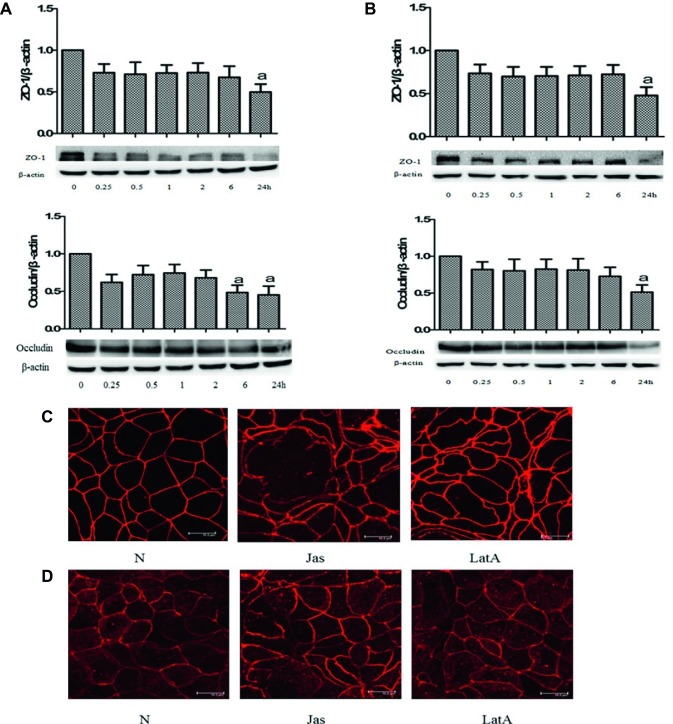
Disruption of actin dynamics insulted tight junction (TJ) proteins. **(A,B)** Caco-2 cells were pretreated with jasplakinolide or latrunculin A (LatA) for 0, 0.25, 0.5, 1, 2, 6, and 24 h, respectively. Cell lysates were analyzed to detect the expressions of zonula occludens (ZO)-1 and occludin. The immunoblot assay showed the relative protein levels of the ZO-1 and occludin after pretreatment with jasplakinolide **(A)** or LatA **(B)**. ^a^*p* < 0.05 compared with 0 h group. **(C,D)** Caco-2 cells were pretreated with jasplakinolide or LatA for 24 h, respectively. The proteins were labeled with Texas red. The cellular distribution of ZO-1 **(C)** and occludin **(D)** along the cell envelope decreased, with morphological changes, such as folds, serrations, and breaks. Scale bar = 10 μm. Data are representative of five similar experiments.

ZO-1 and occludin play important roles in cell tight junction. To clarify whether disruption of actin dynamics can induce a change of TJ proteins, we further detected the protein level of ZO-1 and occludin. The results showed that compared to that at 0 h, the relative expression levels of the ZO-1 and occludin proteins gradually decreased after treatment with jasplakinolide (Figure 4A) or LatA (Figure 4B), with significant reductions in the expression of both proteins at 24 h. It is indicated that imbalance of actin dynamics disrupts cell tight junction by decreasing TJ protein level.

### Jasplakinolide or Latrunculin A Disrupted the Distribution of the Zonula Occludens-1 and Occludin

Based on the above results of TJ protein level, we continued to observe the effect of jasplakinolide or LatA on the distribution of TJ proteins in Caco-2 cells. The results showed that after 24 h of treatment with jasplakinolide or LatA, the morphology of Caco-2 cells became very irregular, with the cell body significantly retracted and gaps forming between the cells. The distribution of ZO-1 (Figure 4C) and occludin (Figure 4D) along the cell envelope decreased, with morphological changes such as folds, serrations, and breaks. It is suggested that disruption of actin dynamics insults TJ *via* TJ proteins.

### Hypoxia Induced Cofilin Activation in Caco-2 Cells

**Figure 5:**
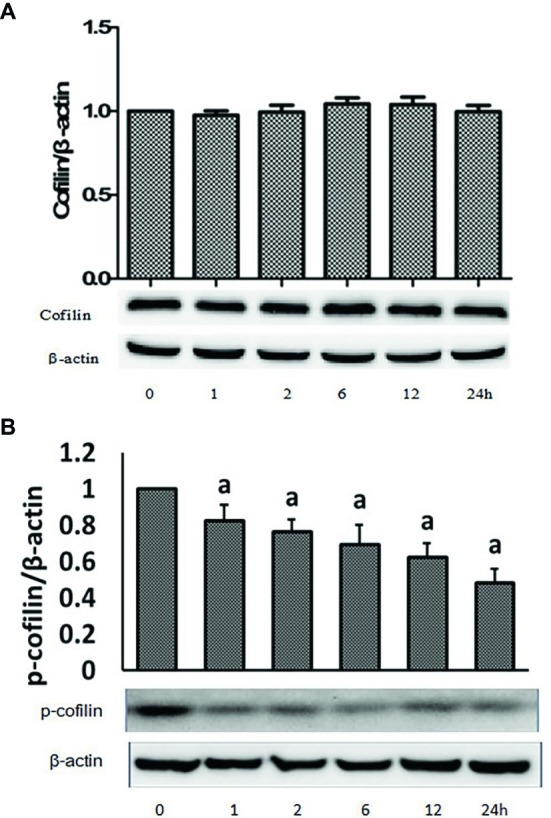
Hypoxia induced cofilin activation in Caco-2 cells. Caco-2 cells were treated with hypoxia for 0, 1, 2, 6, 12, and 24 h, respectively. Cell lysates were analyzed to detect the expressions of cofilin **(A)** and phosphorylated cofilin **(B)** by immunoblot. Hypoxia treatment inhibited the expressions of p-cofilin in a time-dependent manner. ^a^*p* < 0.05 compared with 0 h group. Data are representative of five similar experiments.

The previous studies have shown that the dynamic balance between actin polymerization and depolymerization in cells is mainly regulated by the activity of the cofilin protein. Thus, we further detected the changes in the expression of p-cofilin and total cofilin in Caco-2 cells. As shown in Figure 5A, compared to that before hypoxia (0 h), there were no significant changes in the expression levels of total cofilin protein at different time points after hypoxia. However, as shown in Figure 5B, the expression of p-cofilin protein was reduced to different degrees at different time points after hypoxia, which was significantly lower than that before hypoxia. These results suggested that hypoxia can induce cofilin activation and subsequent disruption of actin networks.

## Discussion

Damage to the intestinal barrier function is an important cause of bacterial translocation, inflammatory responses, sepsis, multiple organ dysfunction, high metabolic responses, and even death after severe burn injury (Carter et al., 2013; Feng et al., 2019). Previous studies have considered that damage to the intestinal barrier function after severe burn injury is mainly the result of the destruction of the histologic structure of the intestinal mucosa. However, in recent years, some scholars have found that the increase in intestinal permeability in the early stages of severe burn injury occurs earlier than the damage to the intestinal mucosa tissue structure. Therefore, the role of intercellular TJs in intestinal epithelial cells and their regulation in intestinal barrier damage in the early stages of burn injuries have received greater attention (Suzuki, 2013; Huang et al., 2018). Our previous studies showed that in the early stages of severe burn injury, the phosphorylation level of the myosin light chain (mediated by the myosin light chain kinase) increased in intestinal mucosal epithelial cells, which played an important role in the occurrence of damage to the intestinal TJ barrier function and the increase in permeability in the early stages of burn injury (Chen et al., 2012). Subsequently, studies by some scholars using the membrane-permeable inhibitor of the myosin light chain kinase (PIK) and myosin light chain kinase gene knockout mice also came to similar conclusions (Guo et al., 2012; Zahs et al., 2012).

To further elucidate the molecular mechanism underlying intestinal TJ barrier damage in the early stages of burn injury, this study investigated the effect of hypoxia on the barrier function of human intestinal epithelial cell monolayers. The TER can be used as a sensitive indicator to evaluate epithelial or endothelial barrier function *in vitro* (Klingberg et al., 2005). We established an intestinal epithelial cell monolayer hypoxia model and selected the TER as an indicator for the evaluation of intestinal epithelial barrier function. Our results showed that the TER significantly decreased after hypoxia, indicating that hypoxia caused damage to the intestinal epithelial cell barrier function. It has been shown that the integrity of the intestinal epithelial cell barrier function mainly depends on the TJ structure between epithelial cells. The molecular anatomical bases of TJs are TJ-related proteins, such as ZO-1, occludin, and claudin-1. Changes in the expression levels of these TJ-related proteins and changes in their intracellular distribution can influence cellular TJs to contribute to barrier dysfunction. Therefore, this study further investigated the effect of hypoxia on the expression and intracellular distribution of TJ proteins, ZO-1 and occludin, in intestinal epithelial cells. The results showed that the expression of these two TJ proteins gradually decreased with increasing hypoxia exposure. The reduction in their expression after 24 h of hypoxia was even more evident. The localization, distribution, and morphology of ZO-1 and occludin in Caco-2 cells after hypoxia significantly changed. There were enormous changes in the cell morphology, and gaps between cells caused by cell body retraction were present. The reduction in expression and changes in distribution of these TJ proteins definitely influenced the structure and function of the TJs between the intestinal epithelial cells, causing damage to the barrier function of the intestinal epithelial cells with reduced TER.

Our previous study showed that changes in F-actin in the intestinal epithelial cytoskeleton might be associated with the occurrence of intestinal barrier damage in the early stages of burn injury (Chen et al., 2012). This study also found that F-actin in Caco-2 cells became disordered after hypoxia and that its density in the cytoplasm was also significantly decreased. In addition, there were occurrences of the hollow phenomenon. These changes indicated that hypoxia induced F-actin depolymerization in cells. It is well known that intracellular F-actin and G-actin maintain a dynamic balance under normal conditions. The destruction of this dynamic balance will induce TJ barrier function damage (Shen et al., 2011; Van Itallie et al., 2015). To further clarify whether the dynamic balance between F-actin and G-actin in intestinal epithelial cells contributed to the intestinal epithelial barrier function damage after hypoxia mediated by TJ-related proteins, drugs that promoted and inhibited actin polymerization were used for intervention. Jasplakinolide is a macrocyclic peptide natural product from sponges. It interferes with actin filaments in the cytoskeleton and plays a regulatory role in the intracellular mechanical processes by influencing actin polymerization/depolymerization (Holzinger and Blaas, 2016; Pospich et al., 2017). LatA binds to G-actin to form a 1:1 complex to prevent the conversion of G-actin into F-actin through polymerization and interfere with microfilament formation in cells (Oliveira et al., 1996). We studied the effect of jasplakinolide or LatA intervention on the expression and distribution of TJ proteins, ZO-1 and occludin, and the TER of Caco-2 cells. The results showed that both jasplakinolide and LatA treatment could induce significant reductions in the expression of the TJ proteins ZO-1 and occludin and could cause significant changes in the distribution of ZO-1 and occludin in the intestinal epithelial cells. In addition, they both could induce a significant reduction in the TER of Caco-2 intestinal epithelial cell monolayers. We considered that the deregulation of the F-actin/G-actin balance in intestinal epithelial cells resulted in the reduction in expression and changes in distribution of TJ proteins to further cause barrier function damage and increase the permeability of the intestinal epithelial cells. A similar study (Van Itallie et al., 2015) showed that after induction of actin depolymerization in renal epithelial cells by LatA, the TJ structure and function of the renal epithelial cells were destroyed, and the barrier function was damaged. The molecular mechanism might be associated with the redistribution of the TJ proteins ZO-1, occludin, and claudin-1 in cells caused by the caveolae-mediated endocytosis of TJ components. Other studies have revealed that several signaling pathways were involved in the cofilin-dependent actin homeostasis, such as EGFR/Src/Akt and RhoA/ROCK pathway (Li et al., 2019; Xue et al., 2019). In addition, work in cultured cells has uncovered the mechanism and regulation of actin-mediated TJ, including Arp2/3 complex (Kang et al., 2013).

Cofilin is an actin depolymerizing factor that can bind to actin in eukaryotic cells. It is currently the only known actin-binding protein that can depolymerize F-actin and plays a very important role in the switch between F-actin and G-actin in cells (Pollard and Borisy, 2003; Grintsevich and Reisler, 2014). To further elucidate the upstream regulatory mechanism underlying the hypoxia-induced deregulation of the dynamic balance between F-actin and G-actin in intestinal epithelial cells, changes in the expression of the cofilin and p-cofilin proteins in Caco-2 cells after hypoxia were further studied. The results showed that there were no significant changes in the expression of total cofilin protein in Caco-2 cells after hypoxia at different time points. By contrast, there was significantly decreased expression of the p-cofilin protein after 24 h of hypoxia. These results suggested that there is hypoxia-induced activation of cofilin in intestinal epithelial cells, which might be the molecular mechanism underlying the deregulation of the dynamic balance between F-actin and G-actin in intestinal epithelial cells.

In summary, severe burn injury causes intestinal epithelial cell barrier function damage. Our results suggest that the molecular mechanism might be that hypoxia activates cofilin and induces F-actin depolymerization and an imbalance between F-actin and G-actin, resulting in a reduction in the expression and a change in the distribution of TJ proteins.

## Data Availability Statement

All datasets generated for this study are included in the article/supplementary material.

## Author Contributions

HS drafted the manuscript. JZ, WH, and PW performed parts of the experiments. FW conceived the experiments and revised the manuscript.

### Conflict of Interest

The authors declare that the research was conducted in the absence of any commercial or financial relationships that could be construed as a potential conflict of interest.
